# Disparate viral pandemics from COVID19 to monkeypox and beyond: a simple, effective and universal therapeutic approach hiding in plain sight

**DOI:** 10.3389/fimmu.2023.1208828

**Published:** 2023-12-01

**Authors:** Howard M. Johnson, Chulbul M. Ahmed

**Affiliations:** Department of Microbiology and Cell Science, University of Florida, Gainesville, FL, United States

**Keywords:** monkeypox virus, coronavirus, antivirals, interferon, SOCS antagonist

## Abstract

The field of antiviral therapeutics is fixated on COVID19 and rightly so as the fatalities at the height of the pandemic in the United States were almost 1,000,000 in a twelve month period spanning parts of 2020/2021. A coronavirus called SARS–CoV2 is the causative virus. Development of a vaccine through molecular biology approaches with mRNA as the inducer of virus spike protein has played a major role in driving down mortality and morbidity. Antivirals have been of marginal value in established infections at the level of hospitalization. Thus, the current focus is on early symptomatic infection of about the first five days. The Pfizer drug paxlovid which is composed of nirmatrelvir, a peptidomimetic protease inhibitor of SARS–CoV2 Mpro enzyme, and ritonavir to retard degradation of nirmatrelvir, is the current FDA recommended treatment of early COVID19. There is no evidence of broad antiviral activity of paxlovid against other diverse viruses such as the influenza virus, poxviruses, as well as a host of respiratory viruses. Although type I interferons (IFNs) are effective against SARS–CoV2 in cell cultures and in early COVID19 infections, they have not been broadly recommended as therapeutics for COVID19. We have developed stable peptidomimetics of both types I and II IFNs based on our noncanonical model of IFN signaling involving the C-terminus of the IFNs. We have also identified two members of intracellular checkpoint inhibitors called suppressors of cytokine signaling (SOCS), SOCS1 and SOCS3 (SOCS1/3), and shown that they are virus induced intrinsic virulence proteins with activity against IFN signaling enzymes JAK2 and TYK2. We developed a peptidomimetic antagonist, based on JAK2 activation loop, against SOCS1/3 and showed that it synergizes with the IFN mimetics for potent broad spectrum antiviral activity without the toxicity of intact IFN molecules. IFN mimetics and the SOCS1/3 antagonist should have an advantage over currently used antivirals in terms of safety and potency against a broad spectrum of viruses.

## Introduction

There is a somewhat Pavlovian response to viral infections that revolves around a very specific approach to development of or identification of antivirals. This approach resides in the chemistry/pharmacology subdiscipline known as medicinal chemistry. Further, there is a penchant for looking for therapeutics via an off-the-shelf approach. Case in point; remdesivir, the broad spectrum pro drug of a nucleotide analog, was approved early by the FDA for treatment of COVID19 in hospitalized patients (1). Remdesivir was originally developed to treat negative stranded RNA Ebola and Marburg virus infections (2). SARS-CoV2 virus is a plus stranded RNA virus, and remdesivir functions as an RNA-dependent RNA polymerase inhibitor, thus it interferes with replication of the virus gene (2, 3). Although FDA approved for treatment of COVID19, it is not clear that remdesivir has much benefit in treating serious illness, particularly regarding moderate to severe disease involving hospitalization beyond early stages of infection (3). On top of this, it can cause liver damage, thus limiting dose and time of treatment (4).

Another drug from the medicinal chemistry tool chest is molnupiravir, a nucleoside analog which like many off-the-shelf antivirals also interferes with SARS-CoV2 RNA-dependent RNA polymerase, but this does not mean that it is specific for SARS (5, 6). It was developed to treat the negative stranded virus of influenza (5). Molnupiravir’s initial metabolite is β-D-N^4^-hydroxycytidine which has been shown to induce mutations in the DNA of mammalian cells (6). This suggests potential cancer-causing properties of molnupiravir. Still, molnupiravir has been shown to be effective in reducing mild, early diagnosed COVID19 from progressing to severe disease and has been approved by the FDA for treatment of non-hospitalized COVID19 patients. The FDA has recommended, however, that molnuparivir should not be the first choice for treatment of COVID19 because of its potential mutational/carcinogenic side effects. In addition, there are the less concerning gut associated potential side effects of nausea, diarrhea, and dizziness (7, 8).

The Pfizer drug paxlovid is the preferred short term treatment of COVID19 as per the FDA. Paxlovid is really composed of two drugs. These are nirmatrelvir, the active ingredient, and ritonavir which slows down the degradation of nirmatrelvir (9). Nirmatrelvir is a peptidomimetic protease inhibitor that binds covalently to the catalytic cysteine residue of SARS-CoV2 protease Mpro (10). Mpro is involved in cleaving the virus polyproteins that results in the production of key virus nonstructural proteins. Paxlovid has shown remarkable effectiveness in a clinical trial involving oral administration within 5 days of onset of COVID19 symptoms with 89 percent reduction in hospitalizations that included COVID19 high risk older patients (9). Like the SARS-CoV2 virus spike protein, mutations have been observed in the Mpro protease (9, 10). While this has not been a clinical problem with respect to virus resistance to paxlovid so far, such resistance has occurred in the use of protease inhibitors in treatment of AIDS which is caused by the human immunodeficiency virus (HIV) (11). One potential way of dealing with this is to use multiple antivirals against the COVID19 virus as was done with AIDS (11). Finally, it should be noted that remdesivir has shown similar protection under similar conditions (3).

The primary innate host defense against viral infections of all types is the interferon (IFN) family of cytokines (12–15). The question arises as to whether the IFN system plays some significant role in host defense against SARS-CoV2 induced COVID19? Focus on this question has resided in types I and III IFNs. SARS-CoV2 is remarkably contagious and the level of mortality/morbidity is unacceptable, at least in most advanced economies. Most SARS-CoV2 infections are asymptomatic or mildly symptomatic, but nonetheless are of concern as these individuals could be contagious (16). If upper respiratory infection does take hold it most likely will result in mild to moderate COVID19 that resolves on its own in healthy individuals. The real concern is severe disease resulting in hospitalization, intensive care, and the potential of debilitating illness and death. It is thought that types I and III IFNs play key roles in the natural host defense that we see in asymptomatic and mild cases of COVID19. While it has been reported that there is no definitive evidence that type I IFNs have any significant benefit in severe COVID19 (17), a recent report describes a unique type I IFN signature in lung neutrophils from severe COVID patients (18) Further, there is no evidence that any of the therapeutics mentioned earlier, including paxlovid with its nirmatrelvir protease inhibitor, have had any significant effect on severe COVID19 (19). With the above reference to nirmatrelvir, it is thus appropriate to look at type I IFNs in terms of their effects on treatment of COVID19 at the early stages of infection. In this regard, IFN-α2b, IFN-β-1a, and IFN-β-1b have shown promise when administered intranasally or injected early in COVID19 infections (20–22).

Recently, monkeypox, a cousin to the smallpox virus and endemic to West Africa, has been reported virtually worldwide (23). Mortality from this virus is low, but its close relationship to smallpox virus warrants attention. The poxviruses are large double-stranded DNA viruses (24), not prone to significant mutations as are coronaviruses like SARS-CoV2. There are monkeypox vaccines and therapeutics in place. This is possibly because of the historical pandemic damage of the smallpox virus which is estimated to have killed 500 million people worldwide since 1900 alone (25). Massive global vaccination in the latter half of the twentieth century eradicated smallpox. There are a variety of tricks employed by the poxviruses to evade host defense in the unvaccinated, probably the most effective being induction of type I and type II IFN decoy receptors (25). Vaccinia virus is a poxvirus that is closely related to variola, the smallpox virus (24). It is highly lethal in mice due to its production of poxvirus B18R type I IFN decoy receptor (25). B18R is illustrative of the simplicity of the mechanism that is the basis of how a virus virulence factor can have a devastating effect on human life. In the IFN mimetic section, we will show how the IFN molecule can be adapted to deal with poxviruses as well as other lethal viruses, including SARS-CoV2 and influenza virus. We will also show that a key immune checkpoint inhibitor system which can function as intrinsic virus virulence factors and composed of a family of intracellular proteins called suppressors of cytokine signaling (SOCS), can be controlled through a simplified concept that results in strong antiviral activity against SARS-CoV2, influenza virus, herpes viruses, picornaviruses as well as a host of other viruses. As indicated above in the title, there is a simple, effective, and universal approach to dealing with a host of virus pathogens; and it is hiding in plain sight. It consists of IFN mimetics and peptidomimetic antagonist of SOCS1/3 (14, 26–28).

## IFN mimetics and IFN signaling

Intrinsically disordered proteins (IDPs) and intrinsically disordered regions (IDRs) in proteins involved in signal transduction have been revealed primarily through the technology of nuclear magnetic resonance (NMR) (29–31). This provided strong support to identification of critical binding sites in proteins in signal transduction that possess the remarkable property of recognition of multiple binding partners at the same time through short continuous and/or contiguous sequences.

Our peptide approach to structure/function predated the discovery that proteins involved in signaling are IDPs and/or proteins with critical IDRs. Our initial approach to studying IFNs via short peptides involved the synthesis of a 20-amino acid peptide corresponding to the N-terminus of human IFNγ (huIFNγ), coupling it to keyhole limpet hemocyanin macroprotein, and using it as an immunogen in rabbits (32). The resultant antibodies interacted specifically with natural huIFNγ, including neutralization of the IFN antiviral activity. The antibodies did not bind to or affect the activity of mouse IFNγ (muIFNγ), huIFNα, or huIFNβ. These results were foundational for the peptide approach to IFN function that has played a central role in our non-canonical model for IFN signaling specifically and cytokine, hormonal signaling in general.

The IFNγ and type I IFN mimetics that have resulted from the model and which are contained in the C-terminus sequences of the IFNs are presented in **Table 1**. The species sources of the IFNs are indicated; however, all IFN mimetics, contrary to their parent IFNs, are species nonspecific. That is, they are all equally active on human and murine cells as well as in their activity in mice (26, 27). MuIFNγ as well as huIFNγ possesses a functional polycationic nuclear localization sequence (NLS) that is required for function and which can be replaced by SV40 virus prototype polycationic NLS, resulting in full recovery of IFNγ antiviral activity (33). MuIFNγ aa (95-133) and huIFNγ aa (95–134) contain the NLSs, KTGKRK for mouse and RKRKRSR for human, in their C-terminus (See **Table 1**) (34, 35). MuIFNγ aa (95–133) was the first IFN mimetic discovered (36). Neither mouse IFNGR1 nor IFNGR2 receptor subunits possess a polycationic NLS (34). IFNGR2 remains on the cell surface while IFNGR1, with attached JAKs, undergoes energy-dependent nuclear translocation in IFNγ or IFNγ mimetic treated cells (35, 36). IFNγ binds primarily to IFNGR1 (35). Endocytosis results in polycationation of IFNγ in the acidic endosomes, resulting in movement to cytoplasm and binding to IFNGR1 cytoplasmic domain (36–38). This increases binding affinity of JAK2 for IFNGR1 and thus movement from IFNGR2 (35). IFNGR2 does not play a further role in signaling. IFNγ NLSs are required for nuclear transport and function of IFNγ as a complex of IFNγ, IFNGR1, activated Janus kinase 2 (JAK2), and activated Signal transducer and activator of transcription α (STAT1α). Details of the nuclear transport are summarized elsewhere (26, 37, 39–41). Importantly, the type I and type II IFN mimetics of **Table 1** are sufficient for nuclear transport of the complexes and resultant IFN function. These mimetics function like their parent IFNs, including the antiviral activity that is not virus specific, as well as antitumor activity in a B16 mouse model of melanoma, and in treatment of experimental allergic encephalomyelitis (EAE), a mouse model of multiple sclerosis (See **Table 1**) (26, 27).

**Table 1:** IFN Mimetics and SOCS1/3 Antagonist.

Peptide Sequence	Anti-		
	viral	Mela noma	EAE
Lipo-huIFNγ aa (95-134): LTNYSVTDLNVQRKAIHELIQVMAELSPAAKTGKRKRSQM	+		
Lipo-muIFNγ aa (95-133): AKFEVNNPQVQRQAFNELIRVVHQLLPESSLRKRKRRC	+		+
Lipo-IFNα1 aa (152-189): LYLTEKKYSPCAWEVVRAEIMRSLSLSTNLQERLRRKE	+	+	+
Lipo-IFNβ aa (150-187): ILHYLKAKEYSHCAWTIVRVEILRNFYFINRLTGYLRN	+		+
Lipo-IFNtau aa (156-195): EKGYS DCAWEIVRVE MRALTSSTTLQ KRLTKTGG DLNSP	+		
Lipo-scrambled: MAVKLGTLNGMYVWLCKSRSTTCGSEDIEVKL EPARQMDTR			
Lipo-pJAK2: ^1001^LPQDKEpYYKVKEP	+	+	
Lipo-pJAK2, scrambled: PEpYVLDKYQKKEP			

Lipo refers to the palmitic acid residue attached to the N terminus for the purpose of gaining entry across the plasma membrane. IFNα1 and IFNβ mimetic sequences are from human IFNs. IFNτ mimetic sequence is from ovine IFNτ. The scrambled peptide is derived by rearranging the sequence of IFNτ mimetic. pJAK2 peptide spans the amino acids 1001 – 1013 from the activation loop of JAK2. Its inactive control is the scrambled pJAK2 peptide. IFN, interferon.

The type I IFN mimetics of **Table 1** were synthesized in terms of the C-terminal region of the parent IFNs using the IFNγ molecule as the template (42, 43). There is a marked difference, however, between these mimetics and the IFNγ mimetics. There is only one IFNγ for the type II receptor complex of IFNGR1/IFNGR2 (35). There may be up to 20 type I IFNs, all operating through the same IFNAR1/IFNAR2 receptor complex with varying degrees of antiviral and apoptotic effects (44, 45). IFNτ was originally identified as an ovine pregnancy hormone. It was later recognized as an IFN with low apoptotic activity (46). Some of the type I IFN mimetics do not possess an obvious putative polycationic NLS (See **Table 1**). Nuclear internalization of these mimetics and their parent IFNβ resides in a functional polycationic sequence in receptor subunit IFNAR1; specifically extracellular sequence ^382^RKIIEKKT of the precursor form of human IFNAR1 (44). Further, both type I receptor subunits IFNAR1 and IFNAR2 undergo active energy dependent nuclear transport complexed to IFN ligand and tyrosine kinase 2 (TYK2) Janus kinase (45). Thus, although IFN mimetic activity resides in the C-terminus of IFNs, there are differences in type I and type II IFN and IFN mimetic signaling in terms of both the ligands and their corresponding receptors. Perhaps related to the diversity of ligands for type I IFN activation relative to a single ligand for IFNGR, it may be evolutionarily advantageous to have the NLS in IFNAR1 rather than in the ligands, given that there are multiple ligands operating through a single receptor. Again, all of these mimetics lack the toxicity associated with the parent IFNs (27, 45). Thus, the relatively simpler IFNγ system has led to our successful discovery of the various type I IFN mimetics.

Viruses have developed a variety of mechanisms to inhibit or blunt the antiviral apparatus of the IFN system. Poxviruses are particularly clever at thwarting the IFN system. These are large double-stranded DNA viruses that replicate in the cytoplasm of the cell. The variola strain of the poxviruses is responsible for smallpox. Worldwide, it is estimated that smallpox has killed up to 500 million people in the 20^th^ century and 400,000 annually in Europe in the 18^th^ century (24). Modern medicine has responded to smallpox by global immunization, but this has been discontinued for many years which has basically resulted in lack of protection of the entire global community to natural or deliberate reintroduction of the virus into society. In addition, the emergence of new poxviruses by recombination within this family of viruses, or by their crossing the species barrier, such as was seen recently in the outbreak with the monkeypox virus from Africa to worldwide are strong reasons for developing effective therapies against this family of viruses.

The assembly of poxviruses in the cytoplasm of infected cells is complex, involving the generation of four types of infectious virus particles. Attachment, internalization, and disassembling of poxviruses is followed by initiation of three waves of mRNA synthesis. The first or early wave codes for virus growth factors and decoy cytokine receptors. Decoy receptors for both type I and type II IFNs are thus produced during early protein synthesis in poxvirus-infected cells. As indicated, the poxvirus decoy receptor for type I IFN is called B18R, while that for type II IFN IFNγ is called B8R (24, 25). Further, smallpox (variola) disease has wracked such havoc on humanity that worldwide immunization has resulted in its eradication by mid twentieth century. If the smallpox virus were to reemerge, we would have a global crisis the likes of COVID19, but with greater mortality and morbidity. This is why the worldwide outbreak of monkeypox, a cousin of smallpox, caused such alarm.

We have shown the mechanism of action of IFN and IFN mimetics (26, 37, 39–41), but we show here that IFN mimetics are quite effective against vaccinia virus in cell culture and in mouse protection. This has implications for treatment of monkeypox. Vaccinia virus, another relative of variola is not harmful to people, but is deadly in a mouse model of lethal poxvirus disease. Intranasal administration of 10^6^ PFU of vaccinia virus (or 10^7^ I.P. administration) results in 100 percent death by day 10. Administration of 20 mg/kg of huIFNα1 aa (152 -189) peptide mimetic I.P. up to 2 days post infection completely protected mice against death (47). It is noteworthy that orally administered muIFNγ aa (95 -133) was also completely protective against vaccinia virus (48). Unlike the parent IFN, the mimetic was nontoxic in mice and in cell culture. The mimetic had a hydrophobic residue (palmitate) attached to facilitate cell penetration. Intact muIFNγ at 5,000 units, in contrast, had no protective effect; all of the mice died by day 10 (47). There is nothing magical about this result. Poxvirus B8R decoy receptor competes with the cell IFNγ receptor, IFNGR1, extracellular domain for IFN. The mimetic binds not to the extracellular IFNGR1 domain but, rather, to the cytoplasmic IFNGR1 binding site, residues 253–287 (26, 37–41). Intact IFNγ after extracellular IFNGR1 binding, binds to the same intracellular IFNGR1 site following endocytosis (26, 37, 39–41). Cell culture experiments with vaccinia virus agree completely with the mouse results (27).

A human type I IFN mimetic, huIFNα1 aa (152–189), was similarly tested in the vaccinia virus mouse model as well as cell culture (27). Conditions were the same as above. As indicated, vaccinia virus like other members of the poxvirus family produces a type I IFN decoy receptor called B18R. HuIFNα1 aa (152–189), administered for the first six days of infection, completely protected mice against a lethal dose of vaccinia, while the intact huIFNα1 was not protective (27). Similar to the IFNγ mimetic, huIFNα1 aa (152–189) was not toxic, causing no loss of weight in contrast to parent huIFNα1 induction of significant weight loss (27). Other type I mimetics (See **Table 1**), along with huIFNα1 aa (152–189), showed similar anti-vaccinia virus inhibition in cell culture, while intact huIFNα1 did not block vaccinia virus replication (27).

IFNγ mimetic muIFNγ aa (95-133) was also active against a highly lethal strain of the picornavirus encephalomyocarditis (EMC) virus in a mouse model with a lethality similar to that of vaccinia virus (49). The type I IFN mimetic huIFNα1 aa (152–189) was also significantly effective in a very aggressive mouse melanoma (B16F10 cells) where untreated mice all died by 20 to 25 days when injected IP with melanoma cells, while mice treated IP with huIFNα1 aa (152–189) for 14 days showed 40 percent survival (27). HuIFNβ has been used extensively as an FDA approved drug in the treatment of relapsing remitting multiple sclerosis. Experimental allergic encephalomyelitis (EAE) is a widely studied mouse model of multiple sclerosis (27). Treatment of EAE with human IFNβ mimetic, huIFNβ aa (150–187), was as effective as the parent IFNβ in ameliorating paralysis (27). As mentioned in **Table 1**, IFNα aa (152-189) and IFNτ aa (156-195) mimetics were also effective in preventing the symptoms of EAE (27). Importantly, the mimetic was not toxic as compared with parent IFN, since treated mice did not suffer weight loss or decrease in lymphocytes (27, 45). In cell culture, the IFN mimetic caused less apoptosis than the natural IFN (45). We have demonstrated previously that a greater affinity of the IFN with its receptor subunit was responsible for greater cell cytotoxicity (46). Since the cell penetrating IFN mimetic does not interact with the extracellular surface of the receptor, that may account for lack of apoptosis in cells in culture (45).

## Development of SOCS1/3 antagonist as a broad antiviral agent

**Figure 1:**
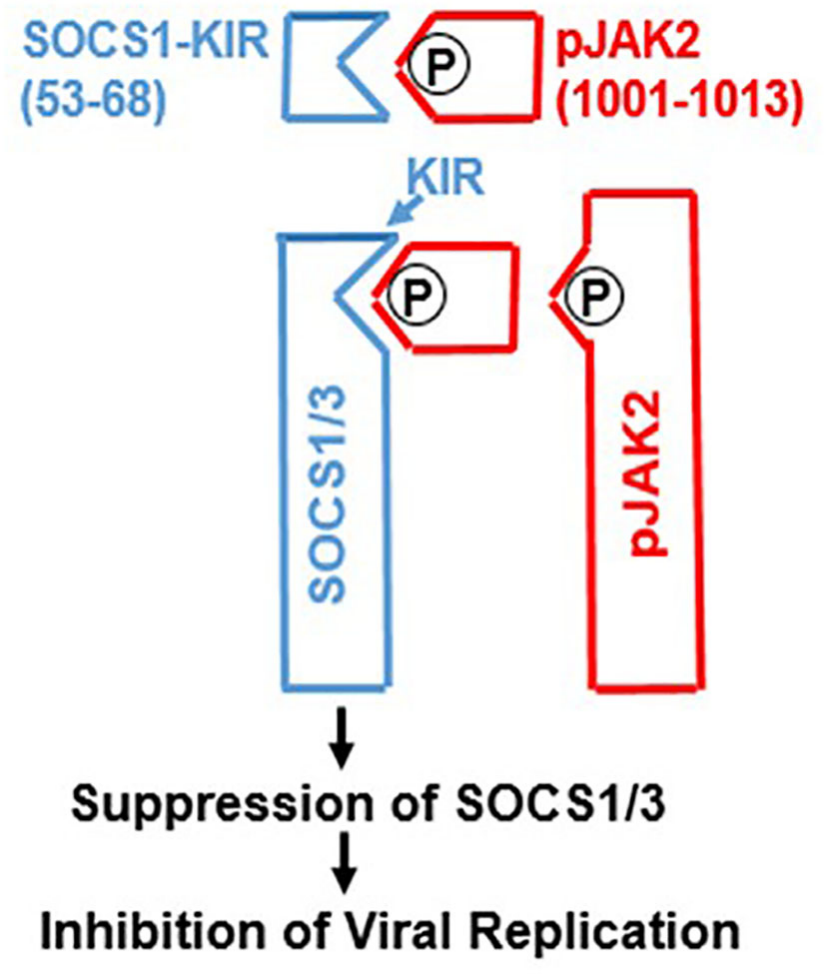
Development of SOCS1/3 antagonist. A peptide spanning amino acids 1001-1013 from the activation loop of tyrosine kinase JAK2, in which tyrosine at 1007 is phosphorylated was synthesized. Attachment of polyarginine (R9) or palmitoyllysine (lipo) to this peptide makes it cell permeable. This peptide binds to the kinase inhibitory region (KIR) of SOCS1 or SOCS3 and thereby allows the activation of JAK2 (or TYK2) for induction of antiviral activity. P, phosphorylation.

The IFN system, including its mimetics, operate in a complex host defense environment. In addition to host protective players like the IFNs, there are the checkpoint inhibitors of the extent of host defense responders such as IFNs. Like their parent IFNs, the IFN mimetics must also contend with these inducible checkpoint inhibitors of host defense. Key checkpoint inhibitors in viral infections are two members of the suppressors of cytokine signaling (SOCS) family. The intracellular SOCS of interest are called SOCS1 and SOCS3 (28, 50, 51). SOCS1 and SOCS3 inhibit IFN signaling, in part, by binding to and inhibiting JAK kinases JAK2 (IFNγ) and TYK2 (types I and III IFNs). SOCS1 and SOCS3 are proteins replete with IDRs, including the N-terminal kinase inhibitory regions (KIRs) (52). We synthesized a short peptide that corresponded to the activation loop region of JAK2 (See **Table 1**) as well as a peptide that corresponded to KIR of SOCS1 and showed that peptide SOCS1-KIR bound to the Janus kinase 2 (JAK2) activation loop (Reviewed in reference (28). Further, we showed that SOCS1-KIR blocked activated JAK2 (pJAK2) function (28). The activated JAK2 has a phosphorylated tyrosine on residue 1007 of JAK2 (pJAK2). We synthesized this more specific peptide with the lipophilic palmitate attached for cell penetration. The aim was to provide a decoy receptor for KIR of SOCS1 and thus block SOCS1 function (28, 53). Indeed, pJAK2 peptide blocked both SOCS1 and SOCS3 function by binding to their KIRs. The events described here are illustrated in **Figure 1**.

**Figure 2:**
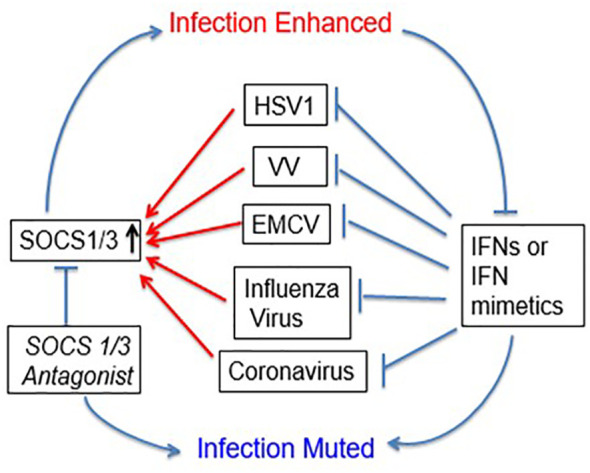
Viral exploitation of host SOCS1/3 to dampen host immune response. Several viruses, including beta coronaviruses have been shown to induce host SOCS1/3 which results in inhibition of signaling through JAK2 and TYK2. A SOCS1/3 antagonist peptide (aka pJAK2) suppresses this activity, resulting in the inhibition of viral replication. IFN mimetic peptide alone, or in synergy with pJAK2 peptide blocks the viral replication.

The development of the SOCS1/3 antagonist had profound inhibitory effects on cellular growth of poxvirus vaccinia virus, vesicular stomatitis virus, small RNA encephalomyocarditis (EMC) virus, herpes simplex virus, influenza virus, as well as coronaviruses including SARS-CoV2 (COVID19) and OC43 that causes common cold (14, 28, 48, 54, 55). Protection against lethal vaccinia virus, EMC virus, and influenza virus in infected mouse models was also observed (28, 48, 54). This protection was due to blockage of the effects of induced SOCS1 and SOCS3 on endogenous IFNs. Exogenous IFNs and IFN mimetics synergized with SOCS1/3 antagonist for enhanced antiviral effects in culture and in mice (14, 28, 48, 55). SOCS1/3 are thus virus induced endogenous virus virulence factors. An example of IFN mimetic and SOCS1/3 antagonist synergism in antiviral activity is illustrated in **Figure 2** (14, 28, 48, 55). In the case of coronavirus OC43, one of the viruses responsible for the common cold, huIFNα1(152 –189) alone significantly inhibited virus induced cell toxicity, consistent with corresponding inhibition of OC43 RNA by inhibition of virus RNA polymerase/helicase activity. SOCS1/3 antagonist had a similar effect; together huIFNα1 aa (152–189) and SOCS1/3 antagonist peptide had a synergistic inhibitory effect on OC43 replication (55). The SOCS1/3 antagonist similarly inhibited replication of SARS-CoV2 RNA gene (55). The IFN mimetic was not tested against SARS-CoV2; however both OC43 and SARS-CoV2 are betacoronaviruses and thus the IFN mimetics and SOCS1/3 antagonist are likely to show synergism against SARS-CoV2. Like other viruses that have been tested, OC43 induces both SOCS1 and SOCS3 (SOCS1/3) in cell cultures (55). These results suggest two things; 1. SOCS1/3 antagonist blocks SOCS1 and SOCS3 inhibition of endogenous IFN in virus infected cells; 2. Addition of IFN mimetics provide excess IFN activity in virus infected cells, adding to that of endogenous IFNs. In addition, SOCS1/3 antagonist was shown to have the following beneficial effects. 1. It enhanced endogenous IFNβ levels; 2. It had adjuvant effects on both cellular and humoral immunity; 3. It was capable of generating immune responses against such poor antigens as bovine serum albumin (56). Likewise, IFNα1 aa (152-189) peptide was also shown to be a strong adjuvant in enhancing both CD4 and CD8 T cell responses (47). A combination of these properties is particularly relevant in the fight against SARS-CoV-2, because it was shown recently that COVID19 infected individuals who were vaccinated and/or exposed to SARS-CoV-2 had a poor response in generating CD8 T cell responses (57). In addition to the viruses mentioned above, there is a number of other viruses of clinical significance that are involved in epidemics/pandemics that induce SOCS1/3 intrinsic virulence factors. Use of the SOCS1/3 antagonist and IFN mimetics should thus prevent and/or blunt the adverse public health effects of these viruses. Viruses in this category include Ebola, Respiratory Syncytial virus, Zika, Dengue and West Nile viruses.

It has recently been reported that individuals immunocompromised with active AIDS are prone to severe monkeypox infection (58). Related to this, a correlation has been shown between upregulation of SOCS1 and SOCS3 as well as PD-1 in dendritic cells and increased HIV plasma load in AIDS patients (59). A similar correlation has been shown in a methicillin-resistant *Staphylococcus aureus* (MRSA) mouse model where skin infection was associated with increased SOCS1 (60). Treatment with a SOCS1 antagonist similar to that described here inhibited SOCS1 and improved host defense against MRSA. We consider these findings preliminary, but suggestive of a role for induced SOCS1 and/or SOCS3 in some forms of immune deficiency. Any evidence of induced SOCS1 and/or SOCS3 playing a role in bacterial infections of immunocompromised terminally ill cancer and non-cancer patients would be of profound importance in controlling these infections and extending lives.

While the paxlovid protease inhibitor nirmatrelvir is currently the FDA preferred therapeutic for early mild COVID19, a phenomenon called rebound COVID19 has been reported in paxlovid treated patients (61). This coupled with the recent demonstration of multiple mutations, with varying degrees of resistance in SARS-CoV2 3CL protease in serial cell cultures of nirmatrelvir and SARS-CoV2 variants, portends virus resistance to this therapeutic over time (62). It would seem, therefore, that our system of IFN mimetics and SOCS antagonists combined with nirmatrelvir e is a logical next step in future studies of SARS-CoV2 variants in COVID19 outbreaks.

## Conclusions

We have discovered and developed a noncanonical model of the mechanism of action of types I and II IFNs. We have further shown a remarkable similarity of this mechanism to that of steroid/steroid receptor signaling. In this regard, we have unified ligand/membrane receptor signaling with that of cytoplasmic/nuclear receptor signaling. Membrane receptor signaling is conceptually dominated by receptor tyrosine kinases and receptor associated tyrosine kinases. The latter in turn is dominated by the canonical model of JAK/STAT signaling pathway. It is our contention that JAK/STAT signaling in the context of this model is incomplete and replete with inexplicable add-on adjustments that have led to such phrases as “The STAT specificity paradox” (63). The central problem is ascribing the specificity of cytokine signaling to STAT transcription factors, and to be inflexible concerning this even in the face of activated JAKs in the nucleus of cells exposed to cytokines and growth factors (37, 64). That activated JAK2 is involved in the key epigenetics of tyrosine phosphorylation on histone H3 for exposure of DNA stirs little attention (65). The types I and II IFN mimetics described here, as per the non-canonical model of IFN signaling have the potential to be effective therapeutics for viral infections, autoimmune disorders, and cancers. Controlled interactions of IFN mimetics and antagonist of the SOCS1/3 checkpoint inhibitors contribute to IFN mimetic activity. Where tested, our mimetics and antagonist, like the FDA-approved peptidomimetic nirmatrelvir, can be effective antiviral and anticancer immunotherapeutics when administered parenterally or orally.

It is our view that their therapeutic value is hiding in plain sight, waiting to be exploited.

## Author contributions

HJ designed the research and wrote the manuscript. CA carried out the experiments. All authors contributed to the article and approved the submitted version.
